# Materials informatics platform with three dimensional structures, workflow and thermoelectric applications

**DOI:** 10.1038/s41597-021-01022-6

**Published:** 2021-09-07

**Authors:** Mingjia Yao, Yuxiang Wang, Xin Li, Ye Sheng, Haiyang Huo, Lili Xi, Jiong Yang, Wenqing Zhang

**Affiliations:** 1 Materials Genome Institute, Shanghai University, Shanghai, 200444 China; 2 State Key Laboratory of Functional Materials for Informatics, Shanghai Institute of Microsystem and Information Technology, Chinese Academy of Sciences, Shanghai, 200050 China; 3 Department of Physics and Shenzhen Institute for Quantum Science & Engineering, Southern University of Science and Technology, Shenzhen, Guangdong 518055 China; 4 Guangdong Provincial Key Lab for Computational Science and Materials Design, and Shenzhen Municipal Key-Lab for Advanced Quantum Materials and Devices, Southern University of Science and Technology, Shenzhen, Guangdong 518055 China

**Keywords:** Thermoelectric devices and materials, Theory and computation

## Abstract

Since the proposal of the “Materials Genome Initiative”, several material databases have emerged and advanced many materials fields. In this work, we present the Materials Informatics Platform with Three-Dimensional Structures (MIP-3d). More than 80,000 structural entries, mainly from the inorganic crystal structural database, are included in MIP-3d. Density functional theory calculations are carried out for over 30,000 entries in the database, which contain the relaxed crystal structures, density of states, and band structures. The calculation of the equations of state and sound velocities is performed for over 12,000 entries. Notably, for entries with band gap values larger than 0.3 eV, the band degeneracies for the valence band maxima and the conduction band minima are analysed. The electrical transport properties for approximately 4,400 entries are also calculated and presented in MIP-3d under the constant electron-phonon coupling approximation. The calculations of the band degeneracies and electrical transport properties make MIP-3d a database specifically designed for thermoelectric applications.

## Abstract (summary)

**Table:** 

Measurement(s)	structural entity • relaxed crystal structure • density of states • band structure • electrical transport property • equation of states
Technology Type(s)	density functional theory • computational modeling technique
Factor Type(s)	material

Machine-accessible metadata file describing the reported data: 10.6084/m9.figshare.15164577

## Background & Summary

Accelerating the process of materials research and development has become a common pursuit in all countries around the world^1,2^. How to quickly obtain new materials based on low-cost and highly reliable prediction methods to guide experiments is an important issue. Traditional materials research and development mainly rely on trial and error, which consumes considerable time and resources. In 2011, the White House proposed the “Materials Genome Initiative” (MGI)^3^. The project consists of three parts: high-throughput (HTP) calculations, experimental methods, and digital databases. Its goal is to reduce the cost of materials research and shorten the development cycle^4,5^.

The MGI focuses on the combination of calculations, experiments, and databases. Among them, big data for materials have been of vital importance. After the proposal of the MGI, several HTP digital material databases based on first-principles calculations have emerged, such as the Materials Project (MP, https://materialsproject.org)^6,7^, the Automatic Flow for Materials Discovery (AFLOW, http://aflow.org)^8–10^, the Open Quantum Materials Database (OQMD, http://www.oqmd.org)^11^, and Novel Materials Discovery (NOMAD, https://www.nomad-coe.eu/)^12^. These platforms all provide basic information such as the formation energy, phase diagram, and electronic structure. They also provide extended functions, including searching for the elastic properties of compounds^13^, piezoelectric materials discovery^14^, online analysis and the design of an algorithm for other material properties. Most of these material databases are open and can be accessed by all researchers seeking to obtain the required material information. These databases have greatly promoted the development of the materials field, for example, thermoelectric materials. Chen *et al*.^15^ studied more than 48,000 materials from MP platform, and calculated the electrical transport properties of approximately 25,000 semiconductor materials to form the MP electrical transport database. Then Ricci *et al*.^16^ made a more detailed summary of the overall distribution of the electrical transport properties in MP. Based on the AFLOW database, Wang *et al*.^17^ calculated the power factors for sintered materials. Toher *et al*.^18^ tested 75 materials based on AFLOW and proposed several low thermal conductivity materials, such as AgI and CuI, which could be used for thermoelectric application.

In recent years, we established our own materials data repository, i.e., the Materials Informatics Platform with Three-Dimensional Structures (MIP-3d). Our initial purpose was to apply big data technology to functional materials (such as thermoelectric materials)^19,20^ of interest. The transport data calculated by home-made packages such as TransOpt^21^ have been integrated into MIP-3d, where electronic relaxation times are computed by the constant electron-phonon coupling approximation (see below). To date, MIP-3d has recorded over 30,000 electronic structures, 4,400 electrical transport properties, and 12,000 equations of state and sound velocities. For entries with finite band gaps, the band degeneracy for the band-edge states has been analysed. Band degeneracy serves as a convenient search criterion for good thermoelectric materials^22^. In the rest of this paper, we present the details of the computational methodology, data record and technical validation of the data in MIP-3d (http://www.mip3d.org).

## Methods

The thermoelectric performance is governed by the dimensionless figure of merit, ZT = (S^2^σT)/κ, where S, σ, T, and κ are the Seebeck coefficient, electrical conductivity, absolute temperature, and thermal conductivity, respectively. In the Boltzmann transport theory, the electrical conductivity σ and Seebeck coefficient S are expressed as follows:

1 $${\sigma }_{\alpha \beta }\left(\mu ,T\right)=\frac{1}{V}\sum _{n{\bf{k}}}{v}_{n{\bf{k}},\alpha }\,{v}_{n{\bf{k}},\beta }{\tau }_{n{\bf{k}}}\left[-\frac{\partial {f}_{\mu }\left({\varepsilon }_{n{\bf{k}}},T\right)}{\partial {\varepsilon }_{n{\bf{k}}}}\right],$$

2 $${S}_{\alpha \beta }\left(\mu ,T\right)=\frac{1}{eTV}{\sigma }_{\alpha \beta }{\left(\mu ,T\right)}^{-1}\,\sum _{n{\bf{k}}}{v}_{n{\bf{k}},\alpha }\,{v}_{n{\bf{k}},\beta }{\tau }_{n{\bf{k}}}\left(\mu -{\varepsilon }_{n{\bf{k}}}\right)\left[-\frac{\partial {f}_{\mu }\left({\varepsilon }_{n{\bf{k}}},T\right)}{\partial {\varepsilon }_{n{\bf{k}}}}\right].$$

Here, ${\varepsilon }_{n{\bf{k}}}$ and **v**_*n***k**_ are the electronic energy and group velocity, respectively, corresponding to band index *n* and reciprocal coordinate **k**, and ${\tau }_{n{\bf{k}}}$ is the electronic relaxation time. $T,\mu ,V,{f}_{\mu },$and *e* are respectively the absolute temperature, the Fermi level, the volume of the unit cell, the Fermi-Dirac distribution, and the electron charge. Identifying high-performance thermoelectric materials by optimizing the individual parameters of ZT is a difficult task^23^. To cope with this challenge, Xing *et al*.^24^ proposed the electronic fitness function *t = (σ/τ)S*^2^*/N*^*2/3*^, where *N* is the volumetric density of states (DOS) and *τ* is the relaxation time. Usually, valley anisotropy^25,26^, band convergence^27^, heavy-light band combinations^28,29^, reduced dimensionality^30^, and nonparabolic bands^31,32^ will complicate the electronic structures and enlarge the fitness function. Good thermoelectric materials usually possess complex electronic structures, and thus, with the help of the electronic fitness function, one can efficiently identify materials with complex band characteristics.

Herein, the electronic relaxation time is the important parameter for determining the electrical transport coefficients. By the full evaluation of the electron-phonon coupling matrix, one can obtain the relaxation time accurately^33,34^, but the computational cost is too high to be applicable in high-throughput calculations. The constant relaxation time approximation can predict the Seebeck coefficient reasonably^35^. However, because of the undetermined relaxation times, the calculations of electrical conductivity are less accurate, which limit the prediction power. Thanks to the constant electron-phonon coupling approximation, the computational cost is moderate and the electrical transport coefficients have been predicted well, such as the studies in diamond-like chalcogenides^20^. The electronic relaxation time in our work is written as:

3 $${\tau }_{n{\bf{k}}}^{-1}=C\,\sum _{n{\prime} {\bf{k}}{\prime} }\delta \left({\varepsilon }_{n{\bf{k}}}-{\varepsilon }_{n{\prime} {\bf{k}}{\prime} }\right).$$

Here *C* is the constant electron-phonon coupling. Equation 3 demonstrates that the electronic scattering phase space is treated explicitly in our method, which is more accurate than the constant relaxation time approximation. The *C* constant can be expressed as follows under the deformation potential approximation:

4 $$C=\frac{2\pi {k}_{B}T{E}_{def}^{2}}{V\hbar G},$$

where *E*_*def*_ is the deformation potential constant of the band edge, and *G* is the Young’s modulus.

Besides the calculations of electrical transport properties, MIP-3d also contains several other quantities suitable for thermoelectric study, such as the band degeneracy and sound velocity. All these calculations make MIP-3d a repository for the HTP study in thermoelectrics. The rest of the work will present the overall workflow and the modules in MIP-3d, as well as the data for thermoelectric-related quantities.

### Workflow

**Fig. 1:**
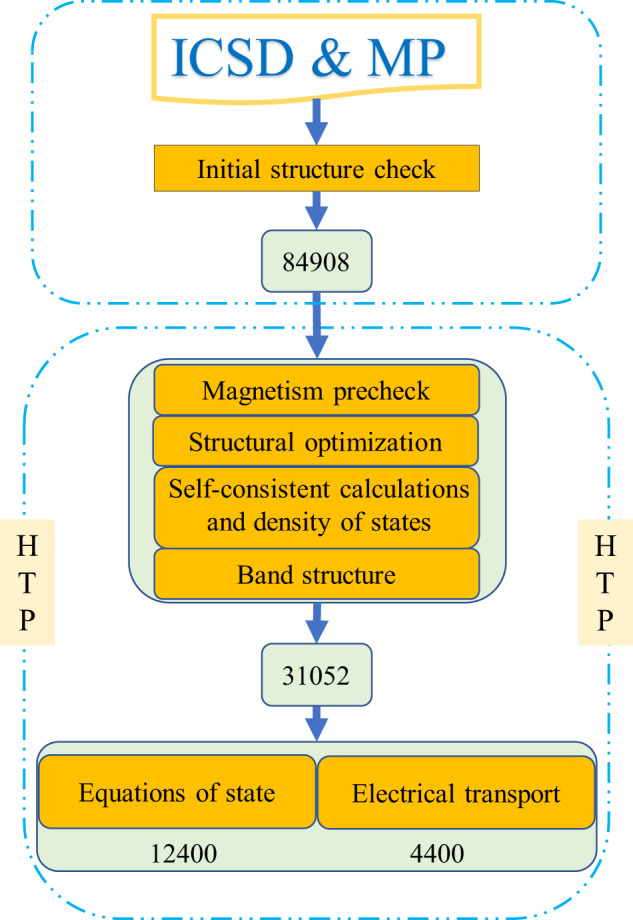
The workflow of MIP-3d. All the statistics are based on calculations up to 2021/01/10.

The calculation method of MIP-3d mainly includes two modules: an initial structure check and HTP calculations. The overall processes are shown in Fig. 1, and each step is explained in detail below.

### Initial structure check

Most of the materials structure information in MIP-3d came from the Inorganic Crystal Structure Database^36,37^ and the MP^6^. All structures with partially occupied atomic sites had been ignored. With the help of the phonopy code^38,39^, we obtained the primitive cells of all compounds, as well as their space groups and the Wyckoff symbols on atomic sites. Duplicated entries were screened out based on the chemical formulas, space groups, and atomic Wyckoff symbols, and we obtained 84,908 entries out of 139,257 initial structures containing 60,628 entries from MP and 78,629 from ICSD.

### High-throughput calculations

We performed first-principles HTP calculations for portions of the 84,908 entries on several of the properties, including structural optimization calculations, self-consistency and DOS calculations, band structure calculations, electrical transport calculations and equation of state calculations. The number of entries for the respective properties is shown in Fig. 1. All the calculations in the present work were performed using the Vienna *ab initio* simulation package (VASP)^40,41^ based on density functional theory with the projector-augmented wave method^42,43^. The Perdew–Burke–Ernzerhof generalized gradient approximation was used as the exchange-correlation functional^44^. The Hubbard U values from ref. ^45^ were applied^46^. In our high-throughput calculation, the same U values were adopted for the same elements in different entries. Recently, Timrov *et al*.^47^ developed a new framework based on the density functional perturbation theory to calculate the U more accurately, but it is not within the scope of this paper. A Gaussian-type smearing with the smearing factor of 0.05 eV was adopted throughout the work. A plane-wave cut-off energy of 520 eV and an energy convergence criterion of 10^−4^ eV for self-consistency were adopted. In this work, most of the pseudopotential files recommended by the VASP (https://cms.mpi.univie.ac.at/vasp/vasp) were adopted, except for W (W instead of W_pv) and Re (Re_pv instead of Re), since some abnormal horizontal lines appeared in the band structures when pseudopotential files of W_pv/Re were used (The comparison of two band structures can be found in the supplemental Fig. S1). The computational parameters and statistics of the respective results are shown below.

#### Magnetism precheck

This module determined whether to set spin-polarization-related tags in the following calculations based on a simple self-consistent calculation with ISPIN=2. The default magnetic moments were 1.0 per atom for ISPIN=2 in VASP. The **k**-point mesh setting in this module was set as (30/|a|+1, 30/|b|+1, 30/|c|+1), where a, b, and c are the lattice parameter values. If the absolute value of the magnetic moment after convergence for the material investigated was greater than 0.02 μB, we tagged this material as spin-polarized and added the line “ISPIN=2” to all the INCAR files for the following calculations. Based on the current statistics, 16,611 compounds were magnetic, and 14,441 compounds were non-magnetic.

#### Structural optimization

The atomic positions, the cell shape, and the volume were relaxed in this module. The **k**-point mesh was set as (30/|a|+1, 30/|b|+1, 30/|c|+1). The convergence criterion of the Hellmann–Feynman force on each atom was less than 10^−2^ eV/Å. For each compound, we initially performed up to 5 VASP rounds of structural optimization with both the atomic positions and cell freely relaxed (ISIF = 3 & IBRION = 2, NSW = 40). If the convergence criterion was not reached, up to 5 more rounds of structural optimization with only the atomic positions relaxed (ISIF = 0 & IBRION = 1, NSW = 40) were conducted. If the compound did not converge after the above ten rounds, it was tagged “relaxation not converged”. Based on the current statistics, 31,052 out of around 33,000 compounds reached the convergence criterion.

#### Self-consistent calculations and density of states

**Fig. 2:**
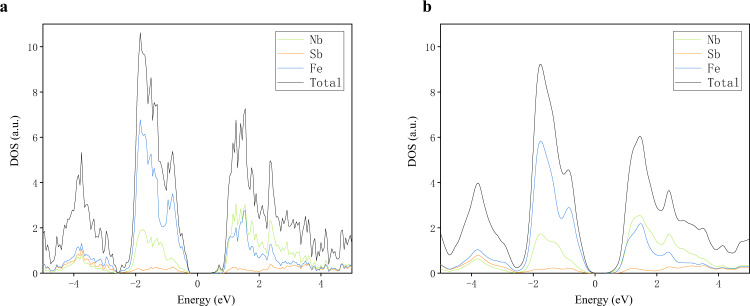
Density of states for MIP3D-17744-Fe1Nb1Sb1 under different levels of smearing.

If the structural optimization was completed with the “converged” tag, the self-consistent calculation was triggered to obtain the charge density, total energy, and magnetic moments (if the material was tagged “spin-polarized” in the magnetism precheck step). The **k**-point mesh used in the self-consistent calculations was (60/|a|+1, 60/|b|+1, 60/|c|+1). Moreover, the projected DOS (as shown in Fig. 2) for the material was also obtained based on the self-consistent calculations, and four plots with different levels of smearing factors are displayed online. In MIP-3d, 31,052 self-consistent calculations, as well as their electronic DOSs, were completed. In some of the subsequent calculations, such as those for the band structures and electrical transport properties, the charge density obtained in this step was adopted.

#### Equations of state

**Fig. 3:**
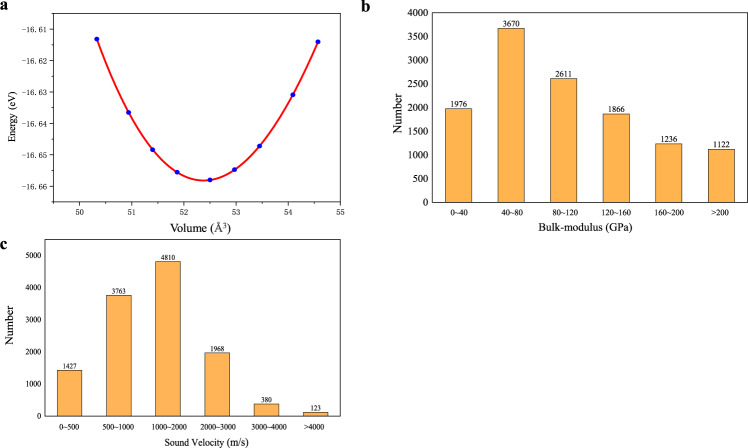
(**a**) The potential surface of entry MIP3D-17744-Fe1Nb1Sb1; (**b**) the distribution of the bulk modulus; and (**c**) the distribution of the sound velocity.

The optimized non-magnetic entries were taken for the equations of state calculations. Nine different volumes, including the optimized volume, were taken into account (Fig. 3a). The structure was scaled to the required volume, and the total energy was subsequently calculated with a self-consistent calculation. The volume-energy potential surfaces was fit by the Vinet-type equation of state to obtain the bulk modulus **K**^48,49^. The 12,400 entries with the fitting determination coefficient R^2^> 0.98 were stored in the MIP-3d database. As shown in Fig. 3(b), for the statistics of **K**, the bulk moduli of most compounds are between 40~120 GPa, which accounts for approximately 50% of the total entries, and approximately 2,000 entries possess **K** values less than 40 GPa. According to the formula ${{\rm{V}}}_{{\rm{S}}}={({\bf{K}}/\rho )}^{1/2}$ (V_S_ is the sound velocity and *ρ* is the density of the compound), a small bulk modulus will result in a low sound velocity of the compound and thus low thermal conductivity^50^. As shown in Fig. 3(c), for the statistics of the sound velocity, 10,000 compounds exist with sound velocities lower than 2,000 m/s, which may be promising in thermoelectric applications.

#### Band structure

**Fig. 4:**
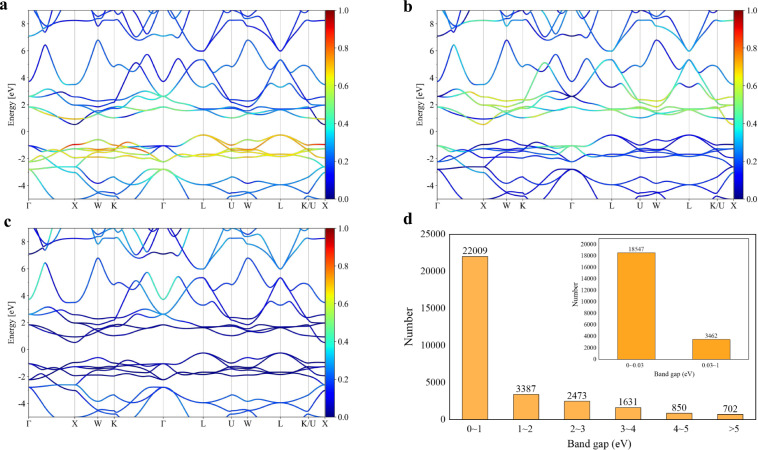
The energy band structure for MIP3D-17744-Fe1Nb1Sb1. (**a**), (**b**), and (**c**) are the elemental projected band structures of Fe, Nb, and Sb, respectively, while (**d**) presents the distribution of the band gap.

The high-symmetry **k**-points of the three-dimensional Brillouin zone used in our band structure calculations referred to refs. ^8,51^. Forty points existed between each pair of high-symmetry **k**-points. Band structure calculations were performed for all the relaxed materials, i.e., 31,052 entries. The band gap values in MIP-3d for all the materials were obtained in this step. As shown in Fig. 4(d), most of the band gaps are below 0.03 eV, for which, in principle, good thermoelectric properties are impossible to achieve. From Table 1, most of the unary (83%), binary (78%), and ternary (60%) compounds in MIP-3d are metallic, while approximately 54% of the quaternary compounds are wider-band-gap (gap > 1 eV) materials. This result suggests that quaternary compounds are more likely to have wide band gaps than unary and binary compounds and shows that the band gap of compounds tends to widen as the number of constituent elements increases.

**Table 1:** Statistical analysis of the band gaps for unary, binary, ternary, and quaternary compounds.

Band gap (eV)	unary	binary	ternary	quaternary
<0.03	434	6,940	9,964	1,138
0.03~1	61	786	1,904	661
1~2	12	481	1,906	915
>2	60	933	3,251	1,236

The numbers in the table denote the number of compounds.

For all the compounds, the elemental projected band structures are displayed, as shown in Fig. 4 for MIP3D-17744-Fe1Nb1Sb1. The bands around the conduction band minima (CBM) for MIP3D-17744-Fe1Nb1Sb1 are typical two-band diagrams, i.e., the CBM at the point X is mainly contributed by Nb, and the second conduction band at the same point is from Fe. The projected DOS plot also reveals the Nb-contributed CBM (Fig. 2); however, the projected band structures are more distinct to demonstrate the band-resolved information. This fact is useful for thermoelectric applications due to the clear presentation of this information, which is lacking in other HTP repositories.

**Fig. 5:**
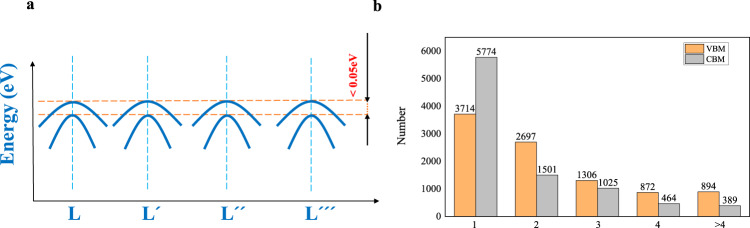
(**a**) Schematic plot of the band diagram-4 band pockets (equivalent **k**-points) with 2 bands in each pocket; (**b**) distribution of the band degeneracy.

The band degeneracy Nv is another useful band-related feature, especially for thermoelectrics^22^. Nv consists of two parts: **k**-point degeneracy and energy degeneracy. The **k**-point degeneracy represents the number of equivalent **k**-points corresponding to one irreducible **k**-point. Within each energy pocket, the number of bands with sufficiently close eigenvalues (0.05 eV from the band edge, either the valence band minima (VBM) or CBM) was defined as the energy degeneracy. A schematic plot of Nv for MIP3D-17744-Fe1Nb1Sb1 is shown in Fig. 5(a). For MIP3D-17744-Fe1Nb1Sb1, the **k**-point degeneracy at VBM (L point) is 4, and the energy degeneracy is 2; thus, the Nv at the VBM of this compound is 8. Band degeneracy is useful for the quick screening of TE materials since a large Nv will result in a large quality factor. We proceeded with Nv analyses for all the materials with band gaps greater than 0.3 eV in MIP-3d. The statistical results of the VBM and CBM are shown in Fig. 5(b). The plot demonstrates the existence of 894 systems with a VBM Nv greater than four. Note that the statistics of Nv are based on the current 0.05 eV criterion for energy degeneracy. If the criterion is set to 0.1 eV, 1,067 entries will have a VBM Nv greater than four.

#### Electrical transport

**Fig. 6:**
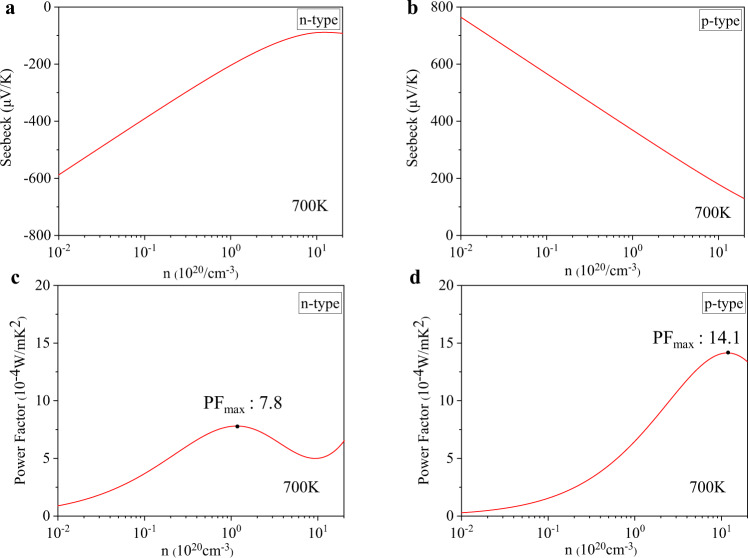
Electrical transport properties at 700 K calculated for MIP3D-17744-Fe1Nb1Sb1. (**a**) and (**b**) present the n-type and p-type Seebeck coefficients, respectively. (**c**) and (**d**) depict the corresponding power factors.

In MIP-3d, for some materials with band gaps > 0.03 eV (more than 4,400), we calculated the electronic transport properties by using TransOpt^21^. A high-density **k**-point mesh (240/|*a*|+1, 240/|*b*|+1, 240/|*c*|+1) was adopted. The electronic group velocity was obtained by the momentum matrix method, as implemented in TransOpt package. The constant electron-phonon coupling approximation was adopted, with the *E*_*def*_ = 3 eV and *G* = 100 GPa for all the materials investigated (Eq. 4). The Seebeck coefficient is independent with the choices of *E*_*def*_ and *G* under the constant electron-phonon coupling approximation, while the electrical conductivity and power factor are relevant to these values. More accurate power factors can be obtained if the HTP deformation potential calculations are to be solved, which will be done in our future work. Fig. 6 shows the calculated electrical transport properties at 700 K for MIP3D-17744-Fe1Nb1Sb1, including the carrier-concentration-dependent Seebeck coefficients and power factors. The choice of temperature 700 K was due to the potential high temperature thermoelectric applications, as also discussed in our previous works^20,21,52^. Based on Fig. 6, the maximum power factors (PF_max_) for both n-type and p-type transport, as well as the corresponding carrier concentrations and Seebeck coefficients, can be obtained.

**Fig. 7:**
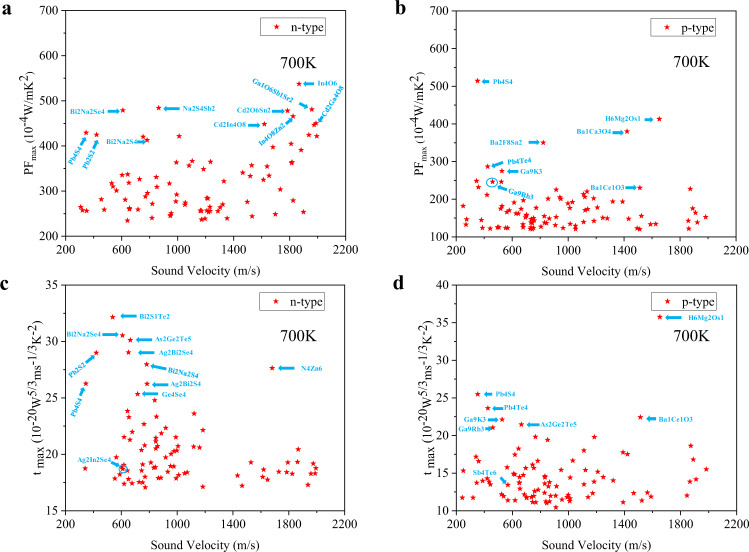
Calculated top 5% maximum power factor and electronic fitness function values along with low sound velocities (<2,000 m/s) for n-type and p-type transport at 700 K. (**a**) and (**b**) are for power factors. (**c**) and (**d**) are for electronic fitness functions.

According to the calculated PF_max_, we took the top 5% of the entries as compounds with promising electrical transport properties. Moreover, a low sound velocity (<2,000 m/s) was taken as the indicator of low thermal conductivity. As shown in Fig. 7(a),(b) and supplemental Table S1, 85 n-type compounds and 90 p-type compounds are screened out. It is a simple screen of the materials with good thermoelectric performance. Although the listed compounds have unusually high absolute values of PF_max_ due to the uniform deformation potential and Young’s modulus, further studies are still worthy due to their good band-related properties. As shown in supplemental Table S1, many chalcogenides and compounds with heavy elements, such as Bi, Pb, are screened out. Furthermore, the maximum electronic fitness functions t_max_ are shown in Fig. 7(c),(d). Due to the fact that the electronic fitness functions have the volumetric DOS in the denominator, the electronic scattering phase space are also considered. By comparing the material suggestions in Fig. 7 (a)–(d), around 40% of the materials screened out by PF_max_ are also recommended by t_max_, implying the similarity of the two methods in proposing new thermoelectric candidates.

## Data Records

Our MIP-3d can be found at http://www.mip3d.org. We have provided output files for all the calculated compounds, which could be found in figshare and our website. A JSON file is available on the web interface (http://mip3d.org/materials/download) and also in a figshare repository^53,54^. Table 2 shows the key variables of the materials database, which include the name, the data type and a short description. ‘id’ is the number of each material in the database. ‘formula’ is the chemical formula, and ‘volume’ is the volume of the unit cell. ‘natoms’ is the total number of atoms in the unit cell, and ‘space_group’ is the space group number of the unit cell. ‘energy’ is the total energy of the system obtained by the static calculation, and ‘is_magnetic’ indicates whether the system is magnetic. ‘total_magnetic_moment’ is the magnetic moment value. In addition, the bulk modulus (‘bulk-modulus’), band gap (‘gap’), and system degeneracy (‘degeneracy_vbm’ and ‘degeneracy_cbm’) are given.

**Table 2:** JSON keys for the data and their descriptions.

Key	Description	Unit or Datatype
**id**	IDs of entries in MIP-3d	string
**formula**	Chemical formula	string
**volume**	Volume of the unit cell	float(Å^3^)
**natoms**	Number of atoms	int
**space_group**	Space group number	int
**energy**	Total energy	float(eV)
**is_magnetic**	Is the material magnetic?	Boolean
**total_magnetic_moment**	The total magnetic moment	float(μB)
**bulk-modulus**	The bulk modulus of this material	float(GPa)
**gap**	Band gap	float(eV)
**degeneracy_vbm**	Band-edge degeneracy for the VBM	float
**degeneracy_cbm**	Band-edge degeneracy for the CBM	float

## Technical Validation

**Fig. 8:**
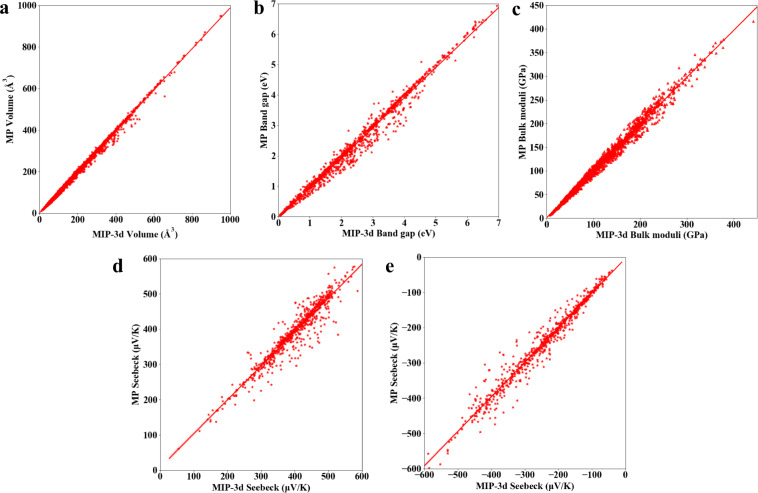
Comparison of the results from MP and those in this work. Comparisons of the volume (**a**), band gap (**b**), bulk modulus (**c**), p-type Seebeck coefficient (**d**) and n-type Seebeck coefficient (**e**). The 0.9 confidence interval is illustrated.

In this work, most of the recommended pseudopotentials from the VASP were adopted, except for W(W) and Re(Re_pv). At each step of the workflow, we set reliable convergence criteria, and the calculation of each step was based on the previous step to achieve convergence. The calculation details were given in the method introduction section above. We performed the following validations for our results in MIP-3d. The Seebeck coefficient values were computed with constant relaxation time (10^−14^ s), 700 K and a doping level of 10^20^ cm^−3^. We benchmarked the volumes (6,000), band gaps (gap > 0.03 eV, 1,100), bulk moduli (1,500) and Seebeck coefficients (739) against the data in an existing 3D material database, MP, as shown in Fig. 8. The Pearson correlation coefficients (the average of the absolute relative errors) between MIP-3d and MP for the volume, band gap, bulk modulus, and Seebeck coefficient are 0.998 (1.71%), 0.991 (6.39%), 0.993 (4.73%), 0.953 (4.59%), and 0.981 (5.39%), respectively, implying high uniformity between this work and MP. Furthermore, we compared the entries with and without U-elements, as shown in the supplemental Fig. S2. The corresponding Pearson correlation coefficients and the average of the absolute relative errors are listed in supplemental Table S2. The Pearson correlation coefficient of band gaps slightly improves from 0.991 (entries with U-elements) to 0.997 (entries without U-elements).

## Usage Notes

In this work, we provided a high-throughput electronic structure database for the prediction and discovery of new materials. Our data can be accessed at www.mip3d.org. In addition, the database is growing rapidly.

## Supplementary information

Supplementary Information

## Data Availability

The calculations of the electrical transport properties in this work rely heavily on TransOpt^21^. The code of TransOpt is available at https://github.com/yangjio4849/TransOpt. In the initial structure checking part, we used the phonopy (http://phonopy.github.io/phonopy/). All the home-made codes used to generate the data is available at https://github.com/yangjio4849/MIP.
