# Supplementary material for: Materials informatics platform with three dimensional structures, workflow and thermoelectric applications
Source: Sci Data. 2021 Sep 7;8:236. doi: 10.1038/s41597-021-01022-6 (PMC8423787; doi:10.1038/s41597-021-01022-6)
Supplement: Supplementary file 1 — Supplementary Information [file 41597_2021_1022_MOESM1_ESM.docx]

**Supplemental Information**

**Materials informatics platform with three dimensional structures, workflow and**

**thermoelectric applications**

Mingjia Yao^1,*^, Yuxiang Wang^1,*^, Xin Li^1^, Ye Sheng^1^, Haiyang Huo^1^, Lili Xi^1^, Jiong Yang^1^, and Wenqing Zhang^2^

1. Materials Genome Institute, Shanghai University, Shanghai 200444, China

2. Department of Physics and Shenzhen Institute for Quantum Science & Engineering, Southern University of Science and Technology, Shenzhen, Guangdong 518055, China

Corresponding authors:

Jiong Yang ([jiongy@t.shu.edu.cn](mailto:jiongy@t.shu.edu.cn))

**Contents of this supplementary file：**

1：Table S1(a), the page numbers of all relevant content is P2-P4.

2：Table S1(b), the page numbers of all relevant content is P5-P7.

3：Figure S1, the page numbers of all relevant content is P8.

4：Figure S2, the page numbers of all relevant content is P9.

5：Table S2, the page numbers of all relevant content is P9

| **name** | **PF_max_ (10^-4^W/mK^2^ )** | **Sound Velocity (m/s)** | **Space group** |
| --- | --- | --- | --- |
| *MIP3D-22204-In4O6 | 537.461 | 1867.23 | 167 |
| MIP3D-26335-Na2S4Sb2 | 484.24 | 864.223 | 12 |
| *MIP3D-18728-Ga1O6Sb1Sr2 | 480.713 | 1958.26 | 225 |
| *MIP3D-7091-Bi2Na2Se4 | 479.012 | 608.282 | 141 |
| *MIP3D-10126-Cd2O6Sn2 | 477.884 | 1784.89 | 148 |
| *MIP3D-22206-In4O8Zn2 | 465.678 | 1824.67 | 227 |
| *MIP3D-10020-Cd2Ga4O8 | 450.081 | 1988.79 | 227 |
| *MIP3D-10059-Cd2In4O8 | 448.407 | 1619.45 | 227 |
| *MIP3D-3015-As2Hg1O6 | 446.493 | 1974.93 | 162 |
| *MIP3D-28773-Pb4S4 | 429.262 | 345.611 | 64 |
| *MIP3D-28725-Pb2S2 | 424.768 | 420.434 | 39 |
| *MIP3D-20220-H2In2O4 | 424.362 | 1937.24 | 31 |
| *MIP3D-28244-O8Sn2Zn4 | 421.637 | 1993.93 | 227 |
| *MIP3D-7593-Br2O2Sb2 | 421.545 | 1010.46 | 129 |
| MIP3D-3056-As2Li2Se4 | 420.154 | 753.418 | 9 |
| *MIP3D-7090-Bi2Na2S4 | 412.919 | 781.572 | 141 |
| MIP3D-27989-O6Sn2Sr2 | 404.667 | 1810.49 | 74 |
| *MIP3D-26078-N4Zn6 | 397.329 | 1680.36 | 194 |
| MIP3D-22189-In4Mg2O8 | 390.845 | 1881.84 | 227 |
| MIP3D-3018-As2Hg2O6 | 366.547 | 1104.15 | 162 |
| MIP3D-18913-Ga2O4Rb2 | 364.891 | 1234.83 | 227 |
| MIP3D-21971-In2Li2O4 | 364.236 | 1816.56 | 141 |
| MIP3D-6191-Ba3Br2In2O5 | 363.498 | 990.659 | 139 |
| MIP3D-21972-In2Li2O4 | 362.336 | 1813.72 | 141 |
| *MIP3D-10267-Cd4O8Sn2 | 356.92 | 1528.88 | 227 |
| MIP3D-6199-Ba3Cl2In2O5 | 356.362 | 1081.12 | 139 |
| MIP3D-843-Ag2O6Sb2 | 354.581 | 1638.97 | 148 |
| *MIP3D-7064-Bi2Li2O4 | 348.396 | 1184.85 | 67 |
| *MIP3D-8939-Ca1Cd3O4 | 340.714 | 1464.77 | 65 |
| *MIP3D-10217-Cd3Se4Zn1 | 337.015 | 642.751 | 215 |
| *MIP3D-10174-Cd2Se2 | 335.428 | 603.191 | 186 |
| MIP3D-10032-Cd2Ge2O6 | 334.064 | 1653.39 | 36 |
| MIP3D-1989-Al2O4Rb2 | 333.191 | 1518.13 | 227 |
| *MIP3D-26336-Na2S4Sb2 | 330.911 | 846.969 | 15 |
| *MIP3D-9916-Cd1Se4Zn3 | 326.419 | 727.643 | 215 |
| *MIP3D-10062-Cd2In4O8 | 324.901 | 1612.55 | 74 |
| MIP3D-756-Ag2In2O4 | 321.765 | 1330.02 | 194 |
| *MIP3D-557-Ag2Bi2Se4 | 318.361 | 651.025 | 141 |
| MIP3D-215-Ag1Cl1 | 317.174 | 528.383 | 225 |
| MIP3D-11414-Cl8Si4 | 316.74 | 941.185 | 19 |
| *MIP3D-7171-Bi2S1Te2 | 310.161 | 537.696 | 12 |
| *MIP3D-7067-Bi2Li2S4 | 309.144 | 851.535 | 141 |
| MIP3D-5232-Ba1Ca1Ga4O8 | 303.59 | 1733.57 | 44 |
| MIP3D-26284-Na2O2Rb2 | 301.013 | 562.636 | 129 |
| *MIP3D-556-Ag2Bi2S4 | 295.475 | 784.305 | 141 |
| *MIP3D-29891-S5Zn5 | 292.206 | 992.767 | 160 |
| *MIP3D-14657-Cu2Se4Sn1Zn1 | 287.947 | 769.38 | 82 |
| MIP3D-502-Ag2As1K1O4 | 284.836 | 1209.1 | 121 |
| MIP3D-9849-Cd1O4Sr3 | 283.463 | 1215.62 | 221 |
| MIP3D-182-Ag1Br1 | 281.62 | 454.487 | 225 |
| MIP3D-16112-F1K1 | 280.844 | 625.245 | 221 |
| MIP3D-1907-Al2K2O4 | 279.146 | 1824.97 | 98 |
| MIP3D-9909-Cd1S4Zn3 | 276.984 | 986.936 | 215 |
| MIP3D-17573-F8Zn4 | 276.055 | 1497.5 | 60 |
| MIP3D-9950-Cd2Cl2H2O2 | 274.329 | 1054.38 | 186 |
| MIP3D-21178-Hg4O4 | 271.77 | 818.948 | 62 |
| MIP3D-16672-F3K1Zn1 | 270.455 | 1322.19 | 221 |
| MIP3D-20528-H6O4Rb2 | 269.706 | 1051.89 | 36 |
| MIP3D-10236-Cd4F8 | 264.94 | 1230.12 | 62 |
| MIP3D-29444-Rb8S4 | 264.742 | 306.987 | 62 |
| MIP3D-1824-Al2F8Rb2 | 263.712 | 1303.86 | 127 |
| MIP3D-6357-Ba4S6 | 261.929 | 655.493 | 109 |
| MIP3D-6356-Ba4S6 | 260.046 | 655.7 | 109 |
| *MIP3D-20012-Ge4Se4 | 259.207 | 716.883 | 62 |
| MIP3D-617-Ag2Cl2 | 258.665 | 475.425 | 63 |
| MIP3D-11138-Cl4O2Rb8 | 257.398 | 319.889 | 122 |
| MIP3D-6897-Bi2Br1Ho1O4 | 256.844 | 1162.06 | 123 |
| MIP3D-6904-Bi2Br1O4Y1 | 256.806 | 1208.62 | 123 |
| MIP3D-6902-Bi2Br1O4Tb1 | 256.497 | 1163.31 | 123 |
| MIP3D-9275-Ca2I10La2 | 256.231 | 349.335 | 63 |
| MIP3D-6894-Bi2Br1Er1O4 | 255.981 | 1169.11 | 123 |
| MIP3D-6942-Bi2Cl1Ho1O4 | 255.786 | 1231.89 | 123 |
| MIP3D-6951-Bi2Cl1O4Y1 | 255.483 | 1287.16 | 123 |
| MIP3D-20459-H4O8P2Rb2 | 253.673 | 1898.94 | 122 |
| MIP3D-16663-F3K1Mg1 | 253.544 | 1220.68 | 221 |
| MIP3D-26449-Na6O3 | 250.787 | 953.477 | 143 |
| MIP3D-698-Ag2Ga2O4 | 248.631 | 1697.8 | 194 |
| MIP3D-16786-F4K2Zn1 | 246.541 | 947.759 | 139 |
| MIP3D-10162-Cd2S2 | 245.166 | 947.595 | 59 |
| MIP3D-1820-Al2F8K2 | 244.055 | 1531.77 | 127 |
| MIP3D-6535-Be2F8Na1Rb3 | 240.449 | 816.62 | 164 |
| MIP3D-4818-B2Na4O6Rb2 | 239.588 | 1353.44 | 59 |
| MIP3D-1355-Al1F6K2Na1 | 238.941 | 1195.4 | 225 |
| MIP3D-7543-Br2H8N2 | 236.755 | 1170.56 | 129 |
| MIP3D-16770-F4H2Rb2 | 234.713 | 641.037 | 140 |

Table S1(a). Calculated top 5% maximum power factor values along with low sound velocities (< 2,000 m/s) for n-type transport at 700 K (‘*’-marked compounds are also recommended by t_max_)

| **name** | **PF_max_ (10^-4^W/mK^2^ )** | **Sound Velocity (m/s)** | **Space group** |
| --- | --- | --- | --- |
| *MIP3D-28772-Pb4S4 | 514.193 | 352.979 | 36 |
| *MIP3D-20513-H6Mg2Os1 | 413.021 | 1651.99 | 225 |
| *MIP3D-5242-Ba1Ca3O4 | 380.052 | 1421.39 | 221 |
| MIP3D-5829-Ba2F8Sn2 | 350.13 | 822.474 | 129 |
| *MIP3D-28775-Pb4Te4 | 286.776 | 425.522 | 62 |
| MIP3D-19176-Ga9K3 | 274.863 | 527.064 | 119 |
| *MIP3D-28773-Pb4S4 | 248.484 | 345.611 | 64 |
| *MIP3D-19180-Ga9Rb3 | 246.432 | 460.605 | 119 |
| *MIP3D-1393-Al1H6In1Rb2 | 246.419 | 523.211 | 225 |
| *MIP3D-167-Ag1Ba2Sb1 | 231.943 | 359.453 | 225 |
| *MIP3D-5249-Ba1Ce1O3 | 230.342 | 1512.94 | 221 |
| *MIP3D-16879-F4O4W2 | 227.381 | 1872.69 | 26 |
| *MIP3D-24322-Li5S4Sb1 | 225.377 | 914.996 | 71 |
| MIP3D-17309-F6Sn2 | 220.445 | 1135.23 | 225 |
| *MIP3D-24362-Li8Mg4Si4 | 213.229 | 1110.34 | 215 |
| *MIP3D-28725-Pb2S2 | 211.446 | 420.434 | 39 |
| MIP3D-9262-Ca2H4 | 206.204 | 1119.59 | 194 |
| MIP3D-11414-Cl8Si4 | 205.121 | 941.185 | 19 |
| *MIP3D-2947-As2Er2O2Zn2 | 202.25 | 1183.09 | 129 |
| *MIP3D-2966-As2F2Sr2Zn2 | 201.326 | 857.303 | 129 |
| *MIP3D-10162-Cd2S2 | 201.03 | 947.595 | 59 |
| MIP3D-6565-Be2Na2Sb2 | 197.021 | 680.235 | 194 |
| *MIP3D-3122-As2O2Y2Zn2 | 193.922 | 1318.95 | 129 |
| *MIP3D-23119-La2Na2O4 | 193.49 | 1389.42 | 141 |
| *MIP3D-30153-Se2Zr1 | 192.521 | 1031.3 | 164 |
| MIP3D-20207-H2He2Li2 | 191.206 | 614.102 | 36 |
| *MIP3D-23144-La2O2Sb2Zn2 | 188.848 | 998.85 | 129 |
| *MIP3D-7661-Br4Cs2Li2 | 181.96 | 250.031 | 129 |
| MIP3D-7356-Bi8I4Ru2 | 181.928 | 527.774 | 87 |
| *MIP3D-28616-P8Th6 | 177.897 | 1209.44 | 220 |
| MIP3D-7542-Br2H4Tb2 | 177.075 | 771.736 | 166 |
| *MIP3D-3484-As8Th6 | 176.782 | 1012.26 | 220 |
| *MIP3D-20514-H6Mg2Ru1 | 175.329 | 1890.17 | 225 |
| MIP3D-13619-Cs2F6Tl2 | 174.908 | 518.629 | 225 |
| *MIP3D-3008-As2Ge2Te5 | 174.431 | 664.142 | 164 |
| *MIP3D-23146-La2O2Te1 | 173.789 | 1140.09 | 139 |
| *MIP3D-2826-As2Be2Li2 | 170.887 | 1121.21 | 129 |
| MIP3D-5643-Ba2Cd2F2Sb2 | 170.259 | 580.254 | 129 |
| *MIP3D-7373-Br1Cl6F1K2 | 165.756 | 562.606 | 225 |
| *MIP3D-17610-Fe1H6Mg2 | 164.06 | 1909.99 | 225 |
| MIP3D-663-Ag2F2Sr2Te2 | 162.199 | 628.314 | 129 |
| *MIP3D-3392-As4Te6 | 162.033 | 644.337 | 12 |
| *MIP3D-2051-Al2Si2Te6 | 158.825 | 702.867 | 162 |
| *MIP3D-2050-Al2Si2Te6 | 156.284 | 700.766 | 147 |
| *MIP3D-6435-Be1H3Na1 | 155.475 | 1523.76 | 221 |
| *MIP3D-6790-Bi1Li5S4 | 154.878 | 829.636 | 71 |
| MIP3D-1396-Al1H6K3 | 152.89 | 914.858 | 139 |
| *MIP3D-6573-Be2P4Si2 | 152.667 | 1983.24 | 122 |
| *MIP3D-29986-Sb2Se2Te1 | 151.716 | 695.117 | 160 |
| *MIP3D-3240-As4Be2Si2 | 150.774 | 1247.89 | 122 |
| *MIP3D-18940-Ga2Se2 | 149.984 | 753.271 | 160 |
| MIP3D-25007-Mg6P4 | 149.136 | 1280.18 | 224 |
| *MIP3D-6579-Be2Sb4Si2 | 148.702 | 865.1 | 122 |
| MIP3D-5564-Ba2Bi1O6Sb1 | 148.116 | 1432.68 | 225 |
| *MIP3D-21975-In2Li2Se4 | 147.29 | 736.542 | 141 |
| MIP3D-7417-Br1O1Rb3 | 146.746 | 275.307 | 221 |
| MIP3D-308-Ag1I1 | 144.785 | 380.457 | 225 |
| *MIP3D-21766-In1Li1Se2 | 144.213 | 758.475 | 166 |
| MIP3D-1876-Al2H6 | 142.77 | 1858.66 | 167 |
| *MIP3D-7064-Bi2Li2O4 | 142.291 | 1184.85 | 67 |
| *MIP3D-5505-Ba1O4Sr3 | 139.647 | 1110.83 | 221 |
| MIP3D-16472-F2H4N2Sn1 | 138.586 | 1923.2 | 12 |
| MIP3D-19085-Ga4Se4 | 137.978 | 735.167 | 187 |
| *MIP3D-9288-Ca2In4P4 | 137.644 | 831.956 | 194 |
| *MIP3D-19084-Ga4Se4 | 137.399 | 744.688 | 194 |
| *MIP3D-10861-Cl2Er2S2 | 136.603 | 844.643 | 59 |
| MIP3D-19076-Ga4S4 | 134.407 | 1014.66 | 194 |
| MIP3D-15663-Er2Li2O4 | 134.224 | 1626.09 | 141 |
| MIP3D-3214-As3Rb1Zn4 | 133.371 | 677.772 | 123 |
| *MIP3D-15662-Er2Li2O4 | 133.363 | 1589.06 | 141 |
| MIP3D-566-Ag2Br4Cs2 | 132.681 | 270.6 | 129 |
| *MIP3D-26327-Na2P2Zn2 | 131.008 | 909.186 | 129 |
| MIP3D-9235-Ca2Ga4P4 | 129.402 | 1052.41 | 194 |
| MIP3D-17032-F6Ga1Li1Tl2 | 127.03 | 806.232 | 225 |
| MIP3D-7181-Bi2Sn1Te4 | 125.702 | 505.356 | 166 |
| MIP3D-2810-As2Ba2Cd2F2 | 125.468 | 678.309 | 129 |
| MIP3D-14208-Cu2F2Sr2Te2 | 125.429 | 735.242 | 129 |
| MIP3D-29395-Rb2Te4Y2 | 125.282 | 495.84 | 194 |
| *MIP3D-3739-Au2Bi2Na4 | 123.962 | 388.12 | 63 |
| *MIP3D-3056-As2Li2Se4 | 123.935 | 753.418 | 9 |
| MIP3D-19096-Ga4Te4 | 123.924 | 553.867 | 194 |
| *MIP3D-3239-As4Ba4Zn2 | 123.795 | 568.138 | 72 |
| MIP3D-27639-O2Sn2 | 123.527 | 964.254 | 31 |
| MIP3D-19842-Ge3O6 | 122.932 | 1493.36 | 152 |
| *MIP3D-5385-Ba1K1P1 | 122.138 | 443.2 | 216 |
| MIP3D-2788-As2B2 | 121.771 | 1862.54 | 186 |
| MIP3D-19170-Ga8Se8 | 121.496 | 729.828 | 186 |
| MIP3D-27849-O4Sn4 | 121.121 | 1053.35 | 36 |
| *MIP3D-22207-In4P4Sr2 | 120.658 | 744.25 | 194 |
| MIP3D-25920-N2S1Th2 | 120.607 | 1511.89 | 164 |

Table S1(b). Calculated top 5% maximum power factor values along with low sound velocities (< 2,000 m/s) for p-type transport at 700 K

Fig. S1. The energy band structure for MIP3D-29490-Re6W2(a, b) and MIP3D-11423-Cl8W2(c, d), a and c were calculated by pseudopotential files of W/Re_pv, b and d were calculated by pseudopotential files of W_pv/Re.


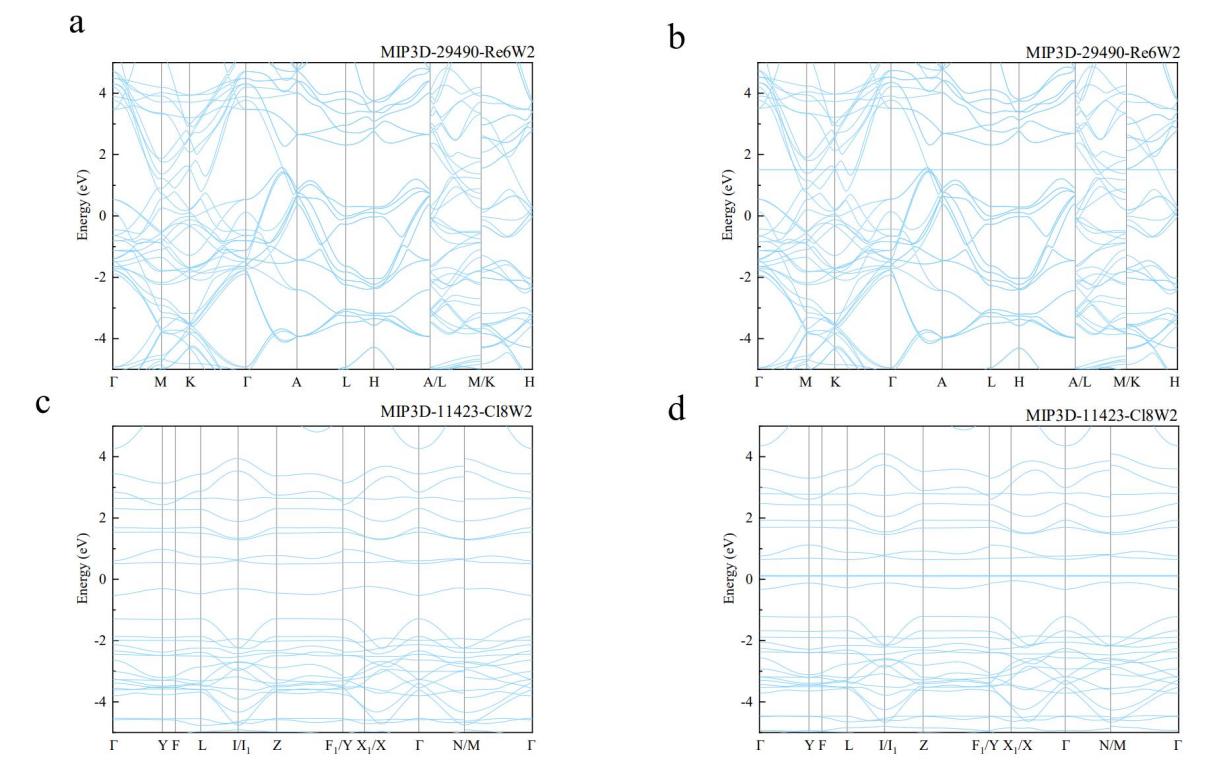


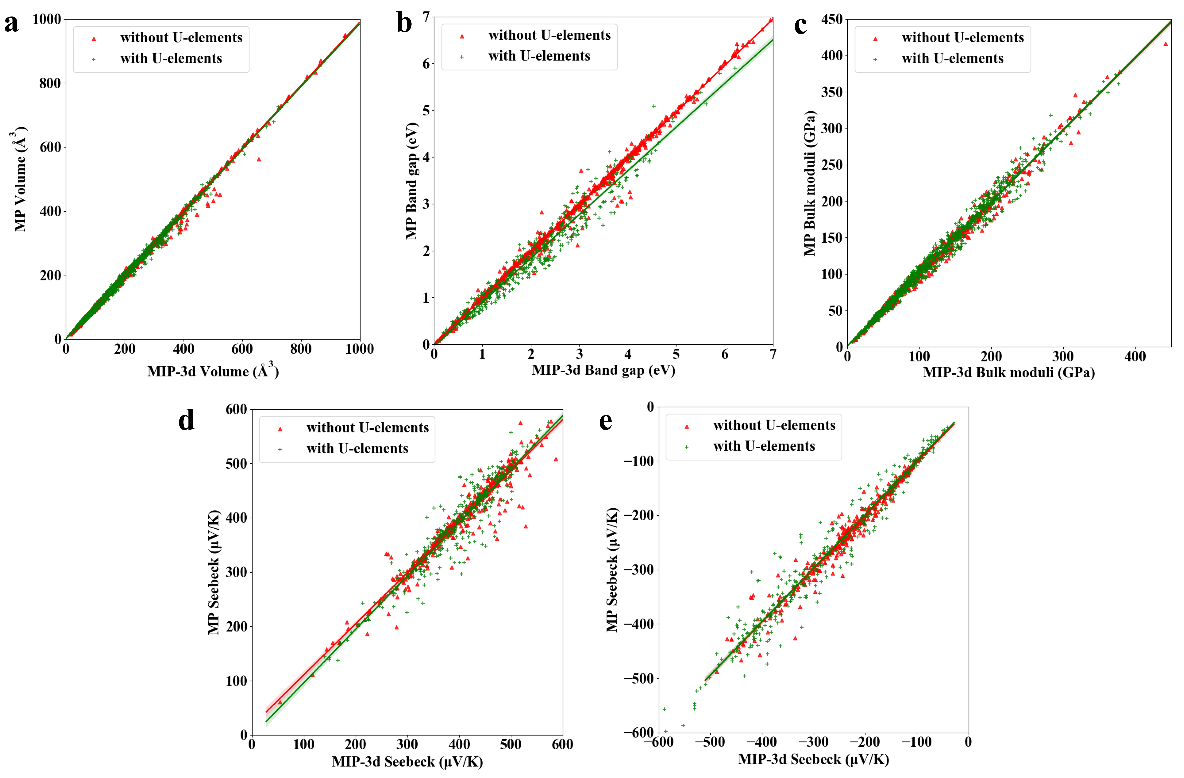


Fig. S2. Comparison of the results from MP and those in this work. Comparisons of the volume (a), band gap (b), bulk modulus (c), p-type Seebeck coefficient (d) and n-type Seebeck coefficient (e). The structures that contain (without) U-elements are plot in green (red). The 0.9 confidence interval is illustrated.

|  |  | | All | with  U-elements | Without  U-elements |
| --- | --- | --- | --- | --- | --- |
| Volume | | Pearson correlation coefficients | 0.9988 | 0.9985 | 0.9988 |
|  |  | the average of the  absolute relative errors (%) | 1.7185 | 2.1139 | 0.8645 |
| Band gap | | Pearson correlation coefficients | 0.9919 | 0.9796 | 0.9972 |
|  |  | the average of the  absolute relative errors (%) | 6.3936 | 11.3869 | 2.8314 |
| Bulk modulus | | Pearson correlation coefficients | 0.9938 | 0.9935 | 0.9945 |
|  |  | the average of the  absolute relative errors (%) | 4.7279 | 4.7887 | 4.5918 |
| Seebeck (p-type) | | Pearson correlation coefficients | 0.9531 | 0.9934 | 0.9657 |
|  |  | the average of the  absolute relative errors (%) | 4.5918 | 4.7887 | 3.1867 |
| Seebeck (n-type) | | Pearson correlation coefficients | 0.9813 | 0.9779 | 0.9853 |
|  |  | the average of the  absolute relative errors (%) | 5.3925 | 7.3641 | 3.4684 |

Table S2. Pearson correlation coefficients and the average of the absolute relative errors.
